# Uptake of dietary milk miRNAs by adult humans: a validation study

**DOI:** 10.12688/f1000research.8548.1

**Published:** 2016-04-22

**Authors:** Amanda Auerbach, Gopi Vyas, Anne Li, Marc Halushka, Kenneth Witwer

**Affiliations:** 1 Department of Molecular and Comparative Pathobiology, The Johns Hopkins University School of Medicine, Baltimore, MD, USA; 2 Department of Pathology, The Johns Hopkins University School of Medicine, Baltimore, MD, USA; 3 Department of Neurology, The Johns Hopkins University School of Medicine, Baltimore, MD, USA

**Keywords:** Milk, miRNA, xenomiR, plasma, PBMC, diet, extracellular vesicles

## Abstract

Breast milk is replete with nutritional content as well as nucleic acids including microRNAs (miRNAs). In a recent report, adult humans who drank bovine milk appeared to have increased circulating levels of miRNAs miR-29b-3p and miR-200c-3p. Since these miRNAs are homologous between human and cow, these results could be explained by xeno-miRNA influx, endogenous miRNA regulation, or both. More data were needed to validate the results and explore for additional milk-related alterations in circulating miRNAs. Samples from the published study were obtained, and 223 small RNA features were profiled with a custom OpenArray, followed by individual quantitative PCR assays for selected miRNAs. Additionally, small RNA sequencing (RNA-seq) data obtained from plasma samples of the same project were analyzed to find human and uniquely bovine miRNAs. OpenArray revealed no significantly altered miRNA signals after milk ingestion, and this was confirmed by qPCR. Plasma sequencing data contained no miR-29b or miR-200c reads and no intake-consistent mapping of uniquely bovine miRNAs. In conclusion, the results do not support transfer of dietary xenomiRs into the circulation of adult humans.

## Introduction

Confirmation that microRNAs (miRNAs) from the diet, or dietary xenomiRs ^1^, could be taken up into mammalian circulation and influence gene expression in the ingesting organism would be a paradigm-shifting development in our understanding of nutrition and cross-organism communication. In animals, mature miRNAs are short oligonucleotides that bind with imperfect complementarity to target sequences in longer RNA molecules including messenger RNAs (mRNAs) ^2^. Loaded into proteins known as Argonautes (AGOs) that recruit additional protein constituents of RNA-induced silencing complexes (RISCs) ^3– 5^, miRNAs may achieve cleavage, translational repression, or sequestration of target molecules, usually resulting in minor post-transcriptional adjustments that fall within the range of normal physiologic variation ^6^, although larger effects have also been described.

Milk is a reported potential source of small RNA molecules in the diet ^7^. The nutritional content of milk includes extracellular vesicles, fat globules, and other structures ^8^, some of which contain miRNAs ^9^. In contrast with plant sources, in which foreign AGO-inferred protection of miRNAs could also preclude uptake and function in mammalian cells, mammalian miRNAs in the diet could be protected by proteins that might function in recipient cells. Baier *et al.* reported transient increases of two miRNAs, miR-29b-3p and miR-200c-3p, in circulating plasma and peripheral blood mononuclear cells (PBMCs) of human donors who drank bovine milk ^10^. In this study of five subjects, blood was drawn at baseline (T0) and at several intervals after milk intake (including T3, T6, T9 and T24). Peak levels of miR-29b and miR-200c were observed at T3 in plasma and at T6 in PBMC. The authors hypothesized that these additional miRNA copies were xenomiRs, supplied by the cow milk as these bovine and human miRNAs are 100% identical. An alternative hypothesis ^11^ suggests that glucose-sensitive endogenous miRNAs, including these two miRNAs ^12– 15^, could respond to dietary intake.

Considering the tremendous implications of these findings for the xenomiR delivery hypothesis, we approached Baier *et al.* and offered to profile additional miRNAs. We hypothesized that more data would help to assess the not necessarily mutually exclusive explanations of the original results: xenomiR delivery or endogenous regulation. The authors generously provided a selection of the original samples. We jointly decided to profile miRNAs at baseline and at the reported peak miRNA concentration for each sample type to look for additional alterations in miRNAs. Accordingly, 10 plasma-derived RNA samples (5 subjects at T0 and T3) and 10 PBMC-derived RNA samples (5 subjects at T0 and T6) were assessed with TaqMan medium-density OpenArray technology arrays and individual miRNA qPCR assays. Finally, these data were compared with small RNA sequencing (RNA-seq) results collected from the same study materials by an independent facility.

## Materials and methods

### Sample delivery, preparation, and quality control

Plasma samples and PBMC total RNA samples, prepared as previously described during an institutional review board-approved study ^10^, were sent to the authors’ laboratory by overnight delivery from the University of Nebraska, Lincoln. No identifying information about the donors was provided. Samples included 10 aliquots of T0 and T3 plasma and 10 T0 and T6 PBMC RNA aliquots from the five participants of the initial study ^10^. Although the package arrived on time via overnight delivery, the dry ice within had largely sublimated. Plasma RNA was isolated using the Exiqon "miRCURY RNA Isolation Kits - Biofluids" (Product # 300112) with modifications as previously described ^16^. PBMC RNA quantity and quality was checked by NanoDrop spectrophotometer (Thermo Scientific). Selected miRNAs were assessed by individual qPCR assays (see below under “qPCR assays”).

### OpenArray profiling and data processing

***A custom miRNA OpenArray chip*** (Thermo Fisher) was designed to detect miRNAs that are consistently found at relatively high abundance in cell-free body fluids such as blood plasma. Features included 220 miRNAs and three non-miRNA small RNAs, RNU44, RNU48, and snRNA U6 (although these are not necessarily suitable references for extracellular RNA). The highly conserved and abundant plant miRNA MIR159a served as a negative control. MIR159a is not expected to be found in mammalian samples, and, if detected, is likely a sign of environmental contaminants ^17, 18^. As expected, MIR159a did not amplify in any sample.

***Sample processing.*** RNA (3 ul plasma RNA or 100 ng PBMC RNA) was reverse-transcribed with the MegaPlex ‘A’ pool of reverse transcription primers (Thermo Fisher). cDNA was pre-amplified using MegaPlex ‘A’ primer pools for a cycle number of 14 (plasma) or 12 (PBMC). Pre-amplified material was diluted per manufacturer’s protocol and as previously described ^19^ and loaded onto the custom TaqMan OpenArray slides by liquid handling robot in the Johns Hopkins DNA Analysis Facility. Quantitative PCR was run for 40 cycles on a QuantStudio instrument.

***Data retrieval and analysis.*** Data including amplification score and relative threshold cycle (Crt) were retrieved using ExpressionSuite software (Thermo Fisher, v1.0.3). Data were analyzed and visualized using Microsoft Excel for Mac 2011, Version 14.5.4, R version 3.2.1, and the Multiple Experiment Viewer (MeV, Version 10.2, part of the TM4 Microarray Software Suite) ^20^.

***Data have been deposited*** with the Gene Expression Omnibus [GEO ^21, 22^, RRID:SCR_004584] under accession numbers GSE79922 (PBMC) and GSE79960 (plasma).

### OpenArray data normalization and analysis

Raw Crt values were normalized by several methods. For plasma, correction factors were calculated based on the geometric mean of the Crts of 22 relatively invariant features (“GM22”) or on Crt of miR-16, a biofluids reference commonly employed in the literature that, while not appropriate in every case, was relatively invariant in these plasma RNA samples. For PBMC, a correction factor was calculated based on the geometric mean of Crt values of 26 features (including the average of U6 readings) that were selected for relatively low variability (“GM26”). A larger number of reference features were selected for the PBMC dataset because of the proportionally larger total number of apparently detected features. A second correction factor was based on the geometric mean of Crt values for the three non-miRNAs on the array, RNU44, RNU48, and the average of U6, which have been used in previous studies as reference RNAs for intracellular RNA expression (“RN3”). Separately, for both plasma and PBMC RNA, quantile normalization was performed, using all RNAs that were detected in at least 9 of 10 samples. Three different adjustments were thus performed for both the plasma and PBMC datasets.

Because of the small number of samples, we performed paired sample t-tests in Microsoft Excel for Mac 2011 v14.5.4 for those miRNAs with complete pre-to-peak sample pairs and considered results potentially significant at p < 0.01.

### Hierarchical clustering

Hierarchical clustering of plasma or PBMC data, filtered by amplification score and for detection in 9 of 10 samples, then adjusted or not via the normalization strategies outlined above, was performed with the Multiple Experiment Viewer v10.2. Data were mean-centered across features. Hierarchical clustering was performed for all samples and features using Pearson correlation and average linkage.

### RNA stability test

Isolated RNA (different plasma source from the above) was aliquoted, frozen at -80 C, and then thawed and left at 22 C for 24, 8, 4, or 0 hours. Quantitative PCR assays (see below) were then used to assess levels of hsa-miR-16-5p and spiked-in cel-miR-39.

### qPCR assays

Stem-loop reverse transcription quantitative PCR assays ^23^ were performed as previously described ^16^. Input was normalized by volume (3 ul for plasma RNA) or mass (33 ng/reaction for PBMC RNA). Assays were ordered from Applied Biosystems/Thermo Fisher, under Inventoried Cat. # 4427975: hsa-miR-1-3p (002222), hsa-miR-16-5p (000391), hsa-miR-125b-5p (000449), hsa-miR-223-3p (002295), hsa-miR-29b-3p (000413), ath-MIR156a (000333), ath-MIR166a (000347), and cel-miR-39 (000200); and ath-MIR167a (000348) under "Made to Order" Cat. # 4440886.

### RNA sequencing data and analysis

RNA sequencing data (BioProject ID PRJNA307561) were downloaded from the Sequence Read Archive and processed on a local server. Analysis was done with miRge [ 24, output: raw and rpm counts for human mapping], Chimira release 1 [ 25, output: raw and normalized by DESeq2 v3.2 ^26^ for human and bovine], and Bowtie v1.1.2 [ 27, RRID:SCR_005476, mapping to human sequences in miRBase v21 ^28^. Sequences were obtained from miRBase v21 for human and bovine comparisons, and milk miRNA profiling data were sought from several previous publications ^9, 29^.

## Results

### Stability of the samples

**Figure 1.**
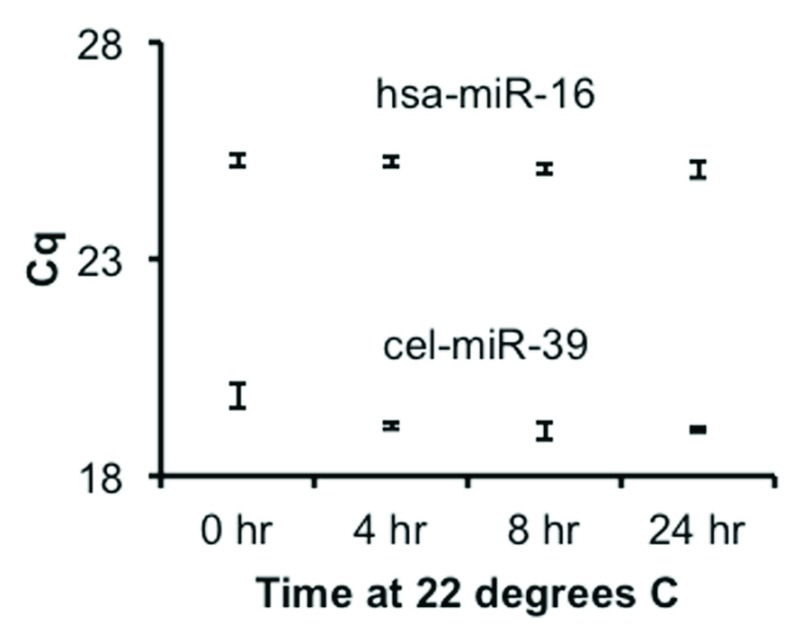
Stability of isolated RNA after thawing. RNA was isolated by the Exiqon Biofluids method from an archived sample of plasma and aliquoted and frozen. Aliquots were thawed and allowed to sit at room temperature (22 C) for 0, 4, 8, or 24 hours. qPCR was used to assess miR-16 and spiked-in cel-miR-39 levels. Shown are +/- 1 standard deviation for triplicate measurements.

The packaging of the shipped samples demonstrated marked sublimation of the dry ice. To assess the stability of the samples, PBMC RNA was measured by spectrophotometer and assessed with stem-loop reverse transcription miRNA qPCR assays for miRs-16, -125b, and -223. This showed limited evidence of degradation, since the signal from each miRNA was consistent across samples ( Table 1) with two exceptions: the zero time point PBMC samples of subjects 4 and 5 amplified 1.9 and 5.8 cycles later, respectively, than the average. There was no indication of general degradation, and it remains unclear whether the two later-amplifying samples were different prior to transport or only after. Plasma stability is less of a concern, since biofluid miRNAs are protected by close association with AGO proteins, whether inside or outside of extracellular vesicles (EVs). Nevertheless, we used qPCR to assess miR-16 stability and that of a spiked-in RNA, cel-miR-39, finding good agreement across samples ( Table 1). Furthermore, RNA samples that were placed at room temperature (22 C) for 0 to 24 hours showed no evidence of degradation of an endogenous or a spiked-in RNA ( Figure 1). We elected to proceed with profiling of all samples, including the two later-amplifying PBMC samples.

**Table 1.** qPCR quality control results. Indicated miRNAs from plasma and PBMC were measured by stem-loop qPCR assays to discover any indications of sample degradation during transport. Shown are average Cq (cycle of quantitation) values across samples as well as standard deviation. For the PBMC samples, removing the two T0 outliers from donors 4 and 5 substantially reduced the variability.

PLASMA			PBMC			
miRNA	Avg Cq	SD	miRNA	Avg Cq	SD	SD-Outliers
miR-16	21.40	0.54	miR-16	22.06	2.64	1.14
Spike	19.84	0.51	miR-125b	32.20	2.10	0.67
			miR-223	21.54	2.41	1.10

### Global analyses of plasma or PBMC miRNAs: OpenArray profiling

***Results overview.*** A total of 223 microRNAs and small RNA controls were assayed by custom qPCR OpenArray, with 134 plasma miRNAs showing amplification in at least 9 of 10 samples. 118 miRNAs were detected in all samples. In RNA from PBMCs, 167 miRNAs amplified in at least 9 of 10 samples, while 135 miRNAs amplified in all samples.

***Analysis criteria.*** We employed an inclusive approach toward identifying miRNAs that differed between baseline (T0) and peak (either T3 or T6) due to the relatively small number of subjects. No p value or false discovery rate corrections were performed. Instead, we flagged as potentially interesting those features that: 1) were detected in at least 9 of 10 samples (and thus supplied at least four of a possible five complete T0-to-peak sample pairs); and 2) had an uncorrected p value by paired t-test of < 0.01. The same criteria were applied to both plasma and PBMC data.

***Plasma miRNA results.*** As shown in Table 2, no plasma miRNAs fulfilled the above inclusion criteria as being altered by milk intake in the raw dataset. Several miRNAs had a nominally significant p value in the normalized datasets. However, there was no overlap between the miRNAs identified in the variously normalized data, indicating a lack of robustness of the findings. These arbitrary findings likely represent type 1 error due to the uncorrected p value cutoff. Furthermore, the average fold change for each feature was less than two fold, with a maximum average upregulation of 92% and a maximum downregulation of 36%.

**Table 2.** miRNAs with uncorrected p < 0.01 in plasma and PBMC samples (OpenArray). Shown are miRNAs that satisfied an arbitrary uncorrected p value (“uncorr. p”) of 0.01 by two-tailed paired t-test, in data normalized variously for plasma: none, GM22 (geometric mean of 22 invariant features), 16 (miR-16), and QN (quantile normalization); and for PBMC: none, GM26 (geometric mean of 26 invariant features), RN3 (geometric mean of U6, RNU44, and RNU48), and QN (quantile normalization). Average fold change (avg FC) across donors is also shown.

PLASMA				PBMC			
Norm	miRNA	Uncorr. p	avg FC	Norm	miRNA	Uncorr. p	avg FC
None	None			None	None		
GM22	miR-636	0.003	0.69	GM26	None		
	miR-92a	0.004	0.69				
16	miR-132	0.002	1.73	RN3	None		
	miR-140	0.007	1.92				
QN	miR-184	0.005	0.64	QN	miR-142-3p	0.005	1.4

***PBMC miRNA results.*** In the PBMC dataset, miR-142-3p was identified in the quantile-normalized dataset, but was not identified under any other normalization strategy ( Table 2). Again, this likely represented type I error. No other miRNAs that differed between time points were identified including those previously evaluated (miR-200c, miR-29b, miR-1) ^1^. Our lack of finding any additionally variant miRNAs due to milk feeding and our inability to replicate the prior study led us to further examine miR-29b and miR-200c.

### Individual analyses of miR-29b and miR-200c

It is possible that expression changes would not reach statistical significance but could still provide some support for the uptake hypothesis, for example, if small increases occurred consistently in all sample pairs or if large but variable fold changes were observed. Therefore, we examined the data for miR-29b and miR-200c for any trends.

***Plasma miR-29b.*** In plasma samples, miR-29b did not amplify consistently and did not satisfy our inclusion criteria above (Crt for only 7 of 10 samples and three complete sample pairs). When we examined these values individually, there was no evidence for intake-related increases. miR-29b appeared to increase slightly for donor 1, but to decrease for donors 2 and 3 (raw values). Following normalization by GM22 or miR-16, there was a decrease in all three complete sample pairs. As for other miRNAs examined, these differences were not statistically significant ( Table 3).

**Table 3.** miR-29b and miR-200c results in plasma and PBMC samples (OpenArray). Results are shown for normalization techniques for plasma: none, GM22 (geometric mean of 22 invariant features), 16 (miR-16), and QN (quantile normalization); and for PBMC: none, GM26 (geometric mean of 26 invariant features), RN3 (geometric mean of U6, RNU44, and RNU48), and QN (quantile normalization). “NA” = not applicable (miRNA data did not satisfy inclusion criteria), “p” = p value (two-tailed paired t-test with no corrections), “FC” = fold change.

PLASMA				PBMC			
Norm	miRNA	p	avg FC	Norm	miRNA	p	avg FC
None	miR-29b	NA		None	miR-29b	0.236	1.51
GM22				GM26		0.331	0.69
16				RN3		0.175	0.57
QN				QN		0.469	0.86
None	miR-200c	0.652	1.09	None	miR-200c	0.050	2.3
GM22		0.367	0.64	GM26		0.927	0.98
16		0.756	0.86	RN3		0.763	0.92
QN		0.277	0.61	QN		0.756	0.95

***Plasma miR-200c.*** miR-200c-associated signal was detected in all plasma samples. This miRNA experienced a slight average downregulation from 0 to 3 hours by 15 to 40% depending on normalization. Although downregulation was observed in four sample pairs (normalized data) or three (raw data), no changes were significant by paired t-test ( Table 3).

***PBMC miR-29b and miR-200c.*** For PBMC samples, the raw miR-29b and miR-200c signals were upregulated by 67% and 93%, respectively, on average ( Table 3). These changes were driven by the donor 4 and 5 T0 samples, which quality control had already identified as having abnormally high Cq values. Following normalization, miR-29b and miR-200c became slightly downregulated.

### Hierarchical clustering reveals within-donor relationships

**Figure 2.**
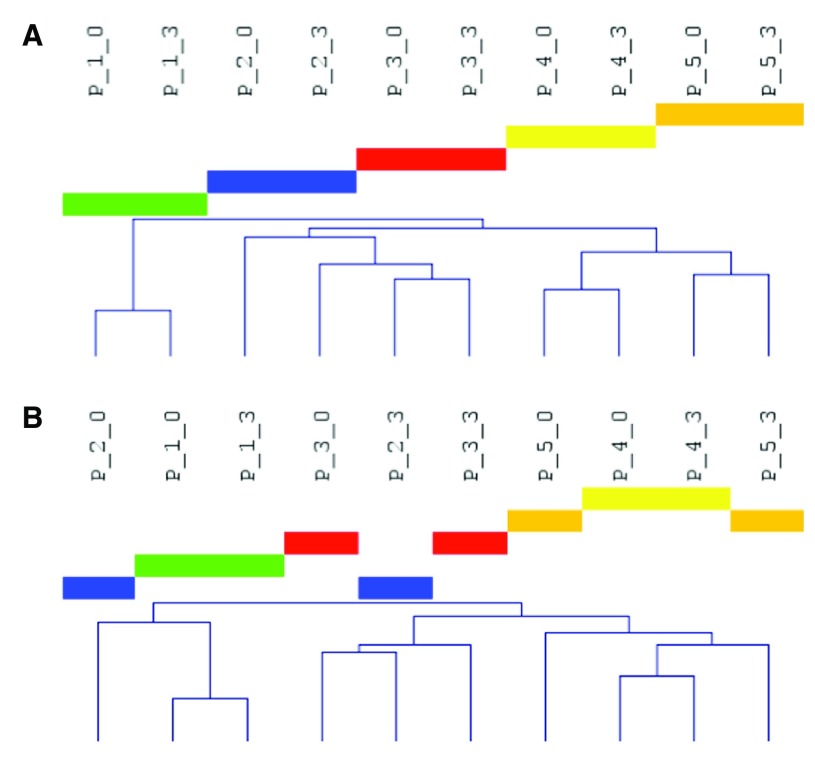
Relationships of plasma samples at T0 and T3. Hierarchical clustering was performed by Pearson correlation and average linkage and conducted for all features from T0 and T3 (previously reported peak of milk-derived RNA expression) using raw data ( **A**) or quantile-normalized data ( **B**) from OpenArray profiling of miRNA. Sample notation “P_1_0” indicates plasma, donor 1, at the 0 hr time point. Each color represents one donor.

**Figure 3.**
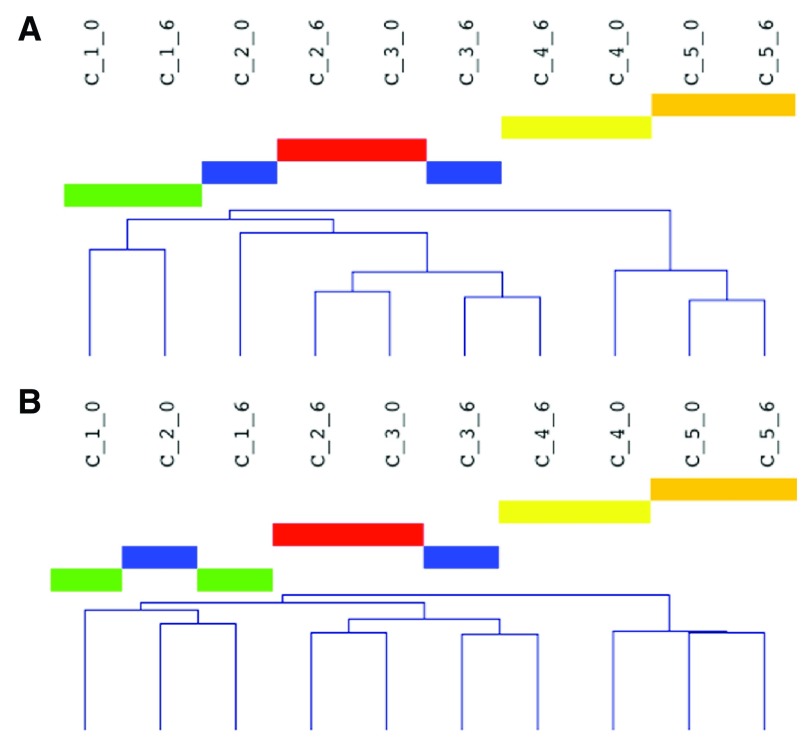
Relationships of PBMC samples at T0 and T6. Sample notation “C_1_0” means cellular (PBMC) RNA, donor 1, at the 0 hr time point. Hierarchical clustering of raw ( **A**) and quantile-normalized data ( **B**) are shown.

Hierarchical clustering was performed to find possible milk-intake-related patterns that were potentially consistent with relationships between samples obtained at the same time points (baseline versus peak) for the entire miRNA dataset. For raw, unadjusted plasma data, samples clustered by donor, not by time point ( Figure 2A). After normalization, no consistent pattern (time point or individual) remained ( Figure 2B; shown is analysis of quantile-normalized data; similar results were obtained from otherwise normalized data, not shown). Similar results were also seen for PBMC data ( Figure 3; as above, results from only raw and quantile-normalized data are shown).

### Individual quantitative PCR assays suggest negligible levels of miR-29b in plasma samples

It is possible that some array-based assays may be less sensitive than standard, higher-volume qPCR assays (10–25 microliters) because of input RNA volume differences. In particular, miR-29b was not detected in all samples, so trends may have been missed. To gather more data on miR-29b if possible, we used individual qPCR assays to probe miR-29b expression and that of known high- and low-expressed miRNAs (miRs-16 and -1). As negative controls, we measured several foreign RNAs (plant miRNAs MIR156a, 166a, and 167a) that are expected to be present negligibly if at all in human plasma. (Also, plant miRNAs are probably not found in milk ^30^.)

**Figure 4.**
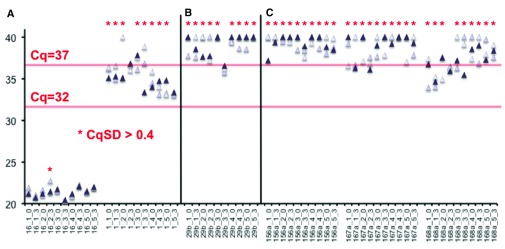
Examples of high, low, and false positive signal detection in plasma RNA samples by quantitative PCR. ( **A**) miR-16 is detected reliably in plasma with Cq in the low 20s, while miR-1 is detected only in the 30s, with high variability. ( **B**) miR-29b-associated signal is detected only late, with no obvious pattern between pre (0) and post (3) milk intake, and is in the same range as spuriously detected plant miRNA-associated signals ( **C**) for ath-MIR156a, -166a, and 167a. * = Cq standard deviation (CqSD) > 0.4. Throughout, the data points are labeled in the format “miRNA_donor_time point”, and replicate measurements are indicated by differently shaded symbols.

***Control miRNAs.***Cq values for miR-16, often used as a control in circulating miRNA studies, were fairly stable across samples, with no obvious pattern of alteration following milk intake ( Figure 4A and Table 1). Only one group of technical triplicates (subject 2, T3) displayed standard deviation of > 0.4 Cq (CqSD). A miRNA normally found at low levels in circulation is miR-1. This largely cardiac and skeletal muscle-specific miRNA may serve as a reliable biomarker of muscle injury precisely because of its normally low abundance in blood ^31^. The low abundance and late detection (all Cq > 32) of miR-1 contributed to large variation in readings even between technical replicates ( Figure 4A, most CqSD > 0.4).

***Low miR-29b signal.*** miR-29b was associated with amplification at even higher Cq values than miR-1, with most replicates amplifying after Cq of 37 and with CqSD > 0.4 ( Figure 4B). miR-29b appears to be present at only very low levels in circulation of these subjects, and its measurement may be subject to the Poisson effect. miR-29b did not appear to change in response to milk intake. Indeed, miR-29b levels are in the same range associated with nonspecific amplification, as shown by non-specific signals from plant miRNA assays. In these samples, three widely conserved plant miRNAs, MIR156a, MIR167a, and MIR166a, had Cq values almost universally later than 37 and with high variability ( Figure 4C).

### RNA-seq analysis: independent dataset

***Analysis of small RNA-seq data.*** It remains formally possible that real responses to milk intake were masked in our studies. Varying degrees of partial degradation of samples that we received could have contributed to the lack of significant differences in our OpenArray results. Compromised samples might also explain the failure to detect substantial amounts of miR-29b by individual qPCR. To determine whether sample integrity or technical problems might have biased our results, we found another data source to analyze. We examined a high-throughput small RNA-seq dataset in the Sequence Read Archive (BioProject ID PRJNA307561) ^32, 33^. In this project, pooled RNA from the same plasma samples we examined was sequenced by an independent facility. The dataset consisted of four pooled samples, representing the five donors at time points 0, 3, 6 and 9 hours post-milk intake. Each sample had between 17.9 and 21 million total reads ( Table 4). We analyzed the sample by three methods: miRge ^24^, Chimira release 1.0 ^25^, and direct mapping via Bowtie v1.1.2 to mature miRNAs. The percent miRNAs were between 96.6 and 99.1% for samples at T0, T6, and T9. However, at T3, the % aligned miRNAs was only 44.9–47%. The dominant miRNA by any measure, representing 43–98% of all miRNAs in these specimens, was miR-486-5p, a miRNA known to be robustly expressed in red blood cells ^34^. Its high level of expression greatly suppressed the RPM counts of all other miRNAs. Despite that, we mapped up to 302 miRNAs, depending on analysis tool and mapping of paralogs. No reads corresponding to miR-29b or miR-200c were detected in any of the samples.

**Table 4.** Mapping percentages by three methods. Total reads in the RNA-seq datasets for plasma at T0, T3, T6, and T9 are shown, along with the number and percentage of reads mapped using miRge, Chimira, or Bowtie.

		miRge		Chimira		Bowtie/miRBase	
SRA	Total reads	mapped	%	mapped	%	mapped	%
SRR3083757	18104234	17571674	97.1	17523557	96.8	17490252	96.6
SRR3083758	21050196	9898504	47.0	9688895	46.0	9442374	44.9
SRR3083759	17972585	17661827	98.3	17620217	98.0	17593830	97.9
SRR3083760	19298056	19119013	99.1	19074396	98.8	19065638	98.8

We additionally evaluated the datasets against bovine miRNAs using Chimira. There are 794 known bovine (bta) miRNAs (miRBase v21), some of which do not appear to have homology to human miRNAs. A total of 205 miRNAs matched to this database, including 165, 131, 138, and 150 miRNAs at 0, 3, 6, and 9 hours. 17 sequences mapped to bta miRNAs without known homologs in humans ( Table 5). Of these, only six had ten raw counts in at least one sample, and only one, bta-miR-1839, was mapped in all samples. However, bta-miR-1839 was most abundant at T0, inconsistent with derivation from milk. The remaining unique bovine miRNA sequences were distributed in a seemingly random fashion amongst the time points.

**Table 5.** Putative bovine-specific miRNAs. Chimira was used to map known bovine miRNAs in RNA-seq data. The miRNAs in this table do not appear to have human homologs. Shown are raw counts at the indicated time points.

miRNA	T0	T3	T6	T9
bta-mir-1839	1333	5	816	157
bta-mir-2338		1	698	
bta-mir-2451	250		1	
bta-mir-2904	4			21
bta-mir-6532		21		
bta-mir-3956	15			
bta-mir-3596	2		3	3
bta-mir-2284z	2	1	1	1
bta-mir-2887	2	1		2
bta-mir-2284x		1	1	1
bta-mir-2484		1	2	
bta-mir-2285z		2		1
bta-mir-2285g		3		
bta-mir-2370-5p	1			
bta-mir-6529b		1		
bta-mir-2892		1		
bta-mir-1388-5p				1

**Milk abundance versus apparent changes at T3.** Since all samples at 0, 3, 6, and 9 hours were pooled, expression comparisons cannot strictly be made. Even so, we compared the miRNAs with the greatest apparent fold increase at T3 with previously reported abundance in milk ^9^. The top ten increased miRNAs were not found in milk or were not among the most abundant in milk ( Table 6). One has no known homolog in cow (hsa-miR-4732-3p). Similarly, the ten miRNAs with the highest reported abundance in milk ^9^ experienced apparent decline or increase with no obvious pattern ( Table 7). This includes let-7b, the most abundant milk miRNA (40% of reads) and also among the most enriched in milk exosomes ^29^, which appeared to decline >100-fold from T0 to T3 (miRge rpm).

**Table 6.** Comparison of plasma miRNAs with reported abundance in milk. Plasma miRNAs with the greatest apparent increase from T0 to T3 by RNA-seq were compared with their reported abundance in milk. The top two increasing miRNAs were not detected (ND) in the initial milk sequencing study ^9^ while another is not found in bovine. The other increasing miRNAs were not among the most abundant in milk.

Greatest increase, T0 to T3		
miRNA	FC (T0 to T3)	Milk rank (ref 9)
208b-3p	9891	ND
126-3p	3177	ND
181b-5p	242	49
183-5p	160	117
125a-5p	148	56
4732-3p	117	ND (human)
181a-2-3p	113	42
141-3p	64	91
423-3p	54	15
100-5p	47	121

**Table 7.** Comparison of milk miRNA abundance with fold change from T0 to T3 in plasma. Of the most highly expressed miRNAs in milk ^9^, some appeared to decline after milk intake, while others increased (“FC” = fold change). let-7d was not detected (ND) in human plasma at the T3 time point.

Top-ranked milk miRNA (ref 9)		
miRNA	% milk miRNAs	FC (T0 to T3)
let-7b	39.1%	-111
let-7a/c	37.0%	5
let-7f	2.8%	14
miR-30a	1.9%	-39
miR-21	1.7%	23
miR-99a	1.7%	-33
let-7d	1.4%	ND (T3)
miR-148a	1.2%	32
miR-92a	1.1%	8
miR-30d	1.0%	-1144

### Discussion

We attempted to replicate and expand on the report of Baier *et al.*, that milk intake increases presence of bovine miRNAs in human plasma and PBMCs. Instead, we found no evidence to support this position. Based on OpenArray analysis and qPCR, we found no evidence of a spike of miR-29b or miR-200c levels at time points 3 and 6 hours for plasma and PBMC, respectively. Additionally, in a screen of 220 miRNAs with multiple normalization methods, we found no consistent evidence of any other miRNA alterations at these time points. We further evaluated pooled small RNA-seq data from these same samples. There was no evidence of any miR-29b or miR-200c in any of these sequencing libraries at any time points based on analysis by three methods of miRNA quantification, despite very low levels of amplification by OpenArray or qPCR in most of these samples. Finally, we found no evidence of unique bovine miRNAs or consistently elevated levels of human/bovine homologs from 3 to 9 hours that would be consistent with dietary uptake.

Although our findings are at odds with Baier *et al.*, they are in agreement with other recent publications. When mouse pups were fed by wild-type dams or transgenic mice engineered to overexpress miR-30b by approximately an order of magnitude in milk ^35^, there were no significant differences in detected miR-30b levels in various organs and blood of nine mice in each group. miR-375 and miR-200c knockout pups experience no significant uptake of these miRNAs from wild-type nursing dams ^36^. As the only experiment to date that can easily distinguish endogenous regulation from exogenous uptake, this study must be given great weight.

### What could explain the discrepancies?

***Normalization.*** Baier *et al.* used a spiked-in RNA as a reference for the plasma miRNA qPCR results. This method does not control for biological variation. Since the spike-in appears to have been used to assign concentrations, the different concentrations in Figure 1 of Baier *et al.* ^10^, could be the result of technical batch effects (see also below) at different time points. Indeed, using this method, the authors have reported miR-29b concentration estimates for bovine milk that appear to range over almost four orders of magnitude, from 20 fmol/L in skim milk (0.2% fat) ^37^ to 50 fmol/L in raw milk, to 150 pmol/L in 1% fat milk ^10^. For PBMC, an endogenous RNA, U6, was used, but this is not a miRNA, and its suitability as a reference was not established in these samples. We would also caution that normalizing late-amplifying (high Cq) features can be misleading.

***RNA purification.*** To obtain RNA from plasma, Baier *et al.* used the NucleoSpin miRNA plasma kit (Machery-Nagel), whereas we used the Exiqon Biofluids kit. We previously reported that the Biofluids protocol outperformed several other methods ^16^, but we have not tested the Machery-Nagel kit. Although the methods might have different recovery efficiencies, it is not clear how this would influence robust findings. Hypothetical RNA purification differences would not affect the PBMC results, since we used the PBMC RNA isolated by Baier *et al.*

***qPCR assay design.*** The qPCR system used in the original study depends on enzymatic addition of sequences to the target miRNA followed by amplification and detection with a sequence-non-specific intercalating dye. The assays we used have sequence-specific RT and qPCR forward primers, as well as a partially sequence-specific hydrolysis probe. It is possible that assays for individual miRNAs might have differing abilities to discriminate between closely-related miRNAs such as members of the miR-29 and miR-200 families.

***Variation.*** In Baier *et al*., the apparent dose- and time-dependence of the results could be within the range of measurement error as no error bars were given. Indeed, establishing less than two-fold expression differences is technically challenging, especially for a small number of samples.

***Sample quality.*** As noted, samples arrived at our laboratory with dry ice largely sublimated. However, as a batch, the data do not indicate poor quality, with the exception of two T0 samples from the PBMC group. Differential degradation of specific samples in transit seems unlikely to have occurred by chance. Thus, these two problematic T0 samples may have been in the same condition in the original study and thereby explain the PBMC miRNA results of Baier *et al.*

***Differential stability of specific molecules in specific samples.*** One might conjecture that the miRNAs detected in plasma at higher concentrations by Baier *et al.* after milk intake were in a particularly labile form that may have been more likely to degrade over time in plasma. However, it is unclear how such degradation could have occurred disproportionately in post-intake plasma.

### Are more studies needed?

***Reproduction.*** An experimental reproduction of the original study might be informative. However, it may be difficult to justify the expense of new studies given that neither our results nor the RNA sequencing echo the original findings. Rigorous transgenic studies have also yielded negative findings ^35, 36^. From the standpoint of stoichiometry, it is also unclear that sufficient miRNA, e.g., miR-29b, is present in milk to achieve the originally reported levels in blood. Based on reported miRNA concentrations, one can calculate the quantity of milk and efficiency of uptake required to achieve hypothetical human plasma alterations. As noted above, the authors’ milk miR-29b estimates have ranged over almost four orders of magnitude ^10, 37^. The lower concentration (20 fmol/L) would necessitate 100% uptake efficiency and stability and at least 75 liters of bolused milk intake to achieve an increase of 300 fmol/L in blood of an ingesting human. At the highest reported concentration of miR-29b, 150 pmol/L, > 1% uptake would be needed. This percentage may be unlikely, as other groups have detected no or negligible dietary miRNA content in an *in vitro* digestion model ^36^ or in intestinal material after gavage of massively non-physiologic quantities of synthetic, modification-stabilized RNA ^38^. Even a purportedly positive study of dietary xenomiRs reported a median uptake of just 0.14% [ 39, not counting a clearly spurious detection ^40^]. While further experimentation would provide additional, welcomed data, it may not be justified by current results or theory.

***Other studies: delivery mechanisms.*** In general, there is a need to learn more about packaging and potential transfer of miRNAs and other cargoes in endogenous EVs or protein complexes. Despite interesting *in vitro* data suggesting transfer of lipophilic dyes from highly concentrated milk EVs to epithelial models ^41^, it is not clear that such experiments are relevant to *in vivo* transfer. In the absence of consistent evidence of xenomiR delivery *in vivo*, studies of delivery mechanisms specific to xenomiRs and their carriers are not strongly indicated.

**Overall conclusion:** miRNA profiling by qPCR array and RNA sequencing of plasma and/or PBMC samples from subjects who ingested milk failed to provide support for uptake of bovine miRNAs. Growing evidence casts doubt on the hypothesis of dietary xenomiR transfer to mammals, whether from different kingdoms ^10, 18, 42, 43^ or related organisms ^35, 36^. Although several related and interesting research questions remain, their exigency is low, and further research in this area should be justified in terms of deliverables for more pressing health questions. In mammals, dietary miRNAs, along with other ingested molecules, are likely to serve as nutrition, not post-transcriptional regulators.

### Data availability

Array data can be accessed in the NCBI’s GEO public repository (RRID:SCR_004584) under accession numbers GSE79922 (PBMC) and GSE79960 (plasma).

Open Science Framework: Dataset: Uptake of dietary milk miRNAs by adult humans: a validation study, doi 10.17605/OSF.IO/M9RQ2 ^44^
