# Supplementary material for: Uptake of dietary milk miRNAs by adult humans: a validation study
Source: F1000Res. 2016 Apr 22;5:721. [Version 1] doi: 10.12688/f1000research.8548.1 (PMC4857747; doi:10.12688/f1000research.8548.1)
Supplement: Peer review history

# Peer review: Referee response for version 1

**DOI:** 10.5256/f1000research.9200.r13517

**Published:** 2016-05-10

**Authors:** Hervé Seitz

**Affiliations:** 1 Institute of Human Genetics (IGH), UPR 1142, French National Center for Scientific Research (CNRS), Montpellier, France

In this article, A. Auerbach and colleagues revisit the published claim that dietary miRNAs can be detected in human blood. Using carefully designed analytical procedures, they show that the original claim was most likely erroneous. Using RNA samples provided by the authors of the initial paper, with various detection techniques and sensitive statistical testing, Auerbach *et al.* could not find any indication of food-derived miRNAs in human plasma or peripheral blood mononuclear cells. They also provide a simple mathematical demonstration that the proposed miRNA dietary intake would require unrealistic volumes of ingested milk. This is a solid manuscript, which addresses an important issue. I only have minor comments: this manuscript is almost already suitable for indexing.

Minor comment #1:

End of the first paragraph of page 2 ("[...] although larger effects have also been described"): bibliographical references are needed here (to my knowledge, large effects were only measured after experimental miRNA over-expression - not with endogenous miRNA levels).

Minor comment #2:

In paragraph "Sample delivery, preparation, and quality control" (page 2): dry ice was "largely sublimated" (that observation is also reported at various other places in the manuscript). Do the authors mean that dry ice was completely gone? Or were there still some pellets? If the styrofoam box is well insulated, just one solid pellet in the box ensures that the atmosphere inside the box is still at -80°C. The authors should at least tell whether RNA solutions were still solid on arrival.

Minor comment #3:

In paragraph "OpenArray data normalization and analysis" (page 2): 26 features were selected for "relatively low variability". How was this done?

Minor comment #4:

In paragraph "RNA stability test" (page 3): was cel-miR-39 spiked-in before or after shipping on dry ice?

Minor comment #5:

The only reliably detected bovine miRNA in human blood is bta-miR-1839, but its accumulation dynamics are not compatible with dietary uptake (first sentence of page 10). The authors may wish to be aware that this "miRNA" is most likely a degradation product from an abundant nuclear RNA, SCARNA15. Full-length bovine and human SCARNA15 are not absolutely identical, but that subsequence (AAGGUAGAUAGAACAGGUCUUGUU) is common to the bovine and human sequences.

I have read this submission. I believe that I have an appropriate level of expertise to confirm that it is of an acceptable scientific standard.

# Peer review: Referee response for version 1

**DOI:** 10.5256/f1000research.9200.r13515

**Published:** 2016-05-04

**Authors:** Jens Allmer

**Affiliations:** 1 Department of Molecular Biology and Genetics, Izmir Institute of Technology, Urla, Turkey

In essence, Auerbach *et al.* tried to replicate findings by Baier *et al*. ^1^ and were unable to come to the same conclusions.

Starting from the alleged (tbk unreproduced) finding of rice miRNAs in human plasma ^2^, a field advocating dietary uptake of xenomiRs and proposing potential regulatory effects has arisen. While the number of studies contesting such findings is rising, it is important that more scrutiny is applied in this context and the study by Auerbach *et al.* is very important in this context.

One clever analysis in this study is the application of clustering to investigate if there is a patient or time point related effect, or none at all. And as it turns out there rather is a patient than a time point effect.

I fully support their conclusion that no more studies in this area are indicated before some additional fundamentals have been established. E.g.: the uptake efficiencies of naked and packed (pre-)miRNAs. Then downstream process like uptake of (pre-)miRNAs by cells and finally the regulatory effect of these need to be established before any further effort in the area of dietary xenomiRs should be exerted.

**Minor Revisions:**

The introduction mentions that ‘essential’ miRNAs would lead to a paradigm shift, but many co-factors and other metabolites like amino acids and vitamins are essential and can only be acquired via food sources. I suggest the statement should be slightly revised and refer more clearly to cross-kingdom communication and less to nutrition.

In Figure 3 the bars marking samples may have a problem in respect to samples 2 and 3 for both panels.

The study by Bagci and Allmer ^3^ does not conclude that there are no plant miRNAs in milk, but rather claims that for the particular samples investigated the plant miRNAs that were found are due to contamination.

However far-fetched this may sound, the Cq values above some threshold are, as pointed out, likely spurious, but I believe that this has to become clear from Figure 4, as well; because some may judge the results as incidence for plant miRNAs in human plasma while they were intended to be negative controls. The figure legend could clearly state that results above Cq X are unreliable.

The methodology describing RNA sequencing data analysis is not reproducible in its current form and could be expanded upon.

Baier *et al*. ^1^ provide error bars for most graphs and present the average +/- SEM (=SD/sqrt(n)) for Figure 1 in Table 1. The section variation in the discussion should be revised accordingly.

xeno-miRNA and xenomiR is used in the text, but xeno-microRNA is not and the abbreviation xenomiR was not introduced as such.

**Questions:**

Why was no spiking applied to the PBMC samples?

Would it be possible to provide the quality assessment of SRA samples to see whether the sample SRR3083758 with the most reads has a much lower sequencing quality than the other samples? Perhaps there is some problem with the file which prevents its proper processing by some of the tools and, therefore, not the complete data was mapped. This needs some clarification.

I have read this submission. I believe that I have an appropriate level of expertise to confirm that it is of an acceptable scientific standard.

## References

[1] BaierSRNguyenCXieFWoodJRZempleniJ: MicroRNAs are absorbed in biologically meaningful amounts from nutritionally relevant doses of cow milk and affect gene expression in peripheral blood mononuclear cells, HEK-293 kidney cell cultures, and mouse livers. *J Nutr*.2014;144(10) : 10.3945/jn.114.196436 1495-500 10.3945/jn.114.196436 25122645PMC4162473

[2] ZhangLHouDChenXLiDZhuLZhangYLiJBianZLiangXCaiXYinYWangCZhangTZhuDZhangDXuJChenQBaYLiuJWangQChenJWangJWangMZhangQZhangJZenKZhangCY: Exogenous plant MIR168a specifically targets mammalian LDLRAP1: evidence of cross-kingdom regulation by microRNA. *Cell Res*.2012;22(1) : 10.1038/cr.2011.158 107-26 10.1038/cr.2011.158 21931358PMC3351925

[3] BağcıCAllmerJ: One Step Forward, Two Steps Back; Xeno-MicroRNAs Reported in Breast Milk Are Artifacts. *PLoS One*.2016;11(1) : 10.1371/journal.pone.0145065 e0145065 10.1371/journal.pone.0145065 26824347PMC4732600

# Peer review: Referee response for version 1

**DOI:** 10.5256/f1000research.9200.r13516

**Published:** 2016-04-29

**Authors:** Cherie Blenkiron

**Affiliations:** 1 Department of Molecular Medicine and Pathology, University of Auckland, Auckland, New Zealand

Here Auerbach *et al* report their attempts to validate the data of Baier *et al *that showed the presence of bovine miRNAs in human plasma samples after ingestion of milk. Baier *et al* kindly provided the original samples for this study but Auerbach *et al* were unable to replicate their findings.

This study is presented in a very thorough and logical manner and the techniques used are appropriate. Multiple analyses are performed in their attempts to validate the findings; they do not rely on a single detection technique or normalisation protocol. The discussion points are also well considered.

Clarity could be provided on the following:

1. The content of the OpenArray. I presume that this was biased towards Human miRNAs. Clarification on the number of miRNAs that are conserved in bovine would be valuable for the reader.
2. In the methods, the OpenArray used a preamplification step but the single probes did not. This was not obvious unless the referenced article was accessed and read. This is a point of difference that affects sensitivity between these assays. With preamplification for the single assays, the Ct values would improve for the low abundance miRNAs and the SD lower for replicates also.
3. The discussion comment on use of differing RNA purification methods might be improved by commenting on why the kits cause differences (e.g. ability to dissociate protein aggregates, lyse EVs etc).
4.  A couple of typos- ul used instead of µL pages 2 and 3. Also a degree symbol is missing on pg 3 for 22 ^o^C.

 In all the authors have presented a well considered and technically robust study that adds further fuel to the debate of cross-species microRNA signaling.

I have read this submission. I believe that I have an appropriate level of expertise to confirm that it is of an acceptable scientific standard.
